# Metachronous port site, muscular and subcutaneous metastases from a gastric adenocarcinoma: a case report and review of articles

**DOI:** 10.1186/s40792-021-01202-x

**Published:** 2021-05-19

**Authors:** Yasuhiro Fukui, Naoshi Kubo, Katsunobu Sakurai, Yutaka Tamamori, Kiyoshi Maeda, Masaichi Ohira

**Affiliations:** 1 Department of Gastroenterological Surgery, Osaka City General Hospital, 2-13-22 Miyakojima-hondori Miyakojima-ku, Osaka City, Osaka 534-0021 Japan; 2 Department of Surgery, Osaka City University Graduate School of Medicine, 1-4-3 Asahi-machi, Abeno-ku, Osaka-City, Osaka 545-8585 Japan

**Keywords:** Gastric cancer, Port site metastasis, Muscular metastasis, Subcutaneous metastasis

## Abstract

**Background:**

Port site metastasis (PSM) after curative gastrectomy for gastric cancer and muscular metastasis from gastric cancer are rare manifestations. Similarly, subcutaneous metastasis from gastric cancer is rare, and muscular and subcutaneous metastases are associated with poor prognosis. We report a case of long-term survival in a patient who underwent curative resection of gastric cancer and repeated recurrence of port site, muscular and subcutaneous metastases from gastric cancer, treated by resection.

**Case presentation:**

A 75-year-old man was diagnosed with gastric cancer and referred to our department. Upper endoscopy demonstrated a 5-cm circumferential ulcerated lesion at the cardia. Biopsy findings showed a poorly differentiated tubular adenocarcinoma. He underwent laparoscopic total gastrectomy with lymph node dissection, and pathologic examination revealed a moderately differentiated tubular adenocarcinoma stage T4aN1M0 and IIIA according to the UICC (Union for International Cancer Control) classification. He refused adjuvant chemotherapy and was only carefully observed. Twenty-three months after the primary gastrectomy, computed tomography (CT) revealed an irregular mass near the port site wounds. Then the patient underwent mass resection, and the pathological diagnosis was consistent with metastatic adenocarcinoma, located in the subcutaneous tissue at the port site wounds. Thirteen months after the second surgery, CT revealed an enhanced mass in the abdominal wall. Positron emission tomography (PET) CT showed an elevated uptake in the rectus abdominis muscle and a standardized uptake value (SUV) of 3.1. The patient underwent another mass resection, and the pathological diagnosis was consistent with metastatic adenocarcinoma in the rectus abdominis muscle. Thirty-five months after the third surgery, CT revealed a mass in the left gluteal subcutaneous region. Furthermore, PET-CT revealed a 35-mm mass with an elevated SUV of 9.6. Another mass resection procedure was performed, and the pathological diagnosis was consistent with metastatic adenocarcinoma in the subcutaneous tissue. Since tumor cells were present at the resection margin, additional radiation therapy was performed. The patient has survived 78 months after primary gastrectomy.

**Conclusion:**

The prognosis of muscular and subcutaneous metastases from gastric cancer is poor. However, if the metastatic tumor is solitary, surgical excision could be a feasible treatment option and might prolong survival.

## Background

The common sites of recurrence and metastasis from gastric cancer are the lymph nodes, peritoneum, and liver. Port site metastasis (PSM) is extremely rare. Muscular metastasis from gastric cancer is a rare manifestation as well. Subcutaneous metastasis from gastric cancer is also rare, with a reported incidence of 0.8–1.0% [1–3]. Both muscular and subcutaneous metastases from gastric cancer are associated with a poor prognosis. Hence, we report a case of long-term survival in a patient who underwent curative resection of gastric cancer and repeated recurrence of port site, muscular and subcutaneous metastases from gastric cancer, which were treated by resection.

## Case presentation

**Fig. 1:**
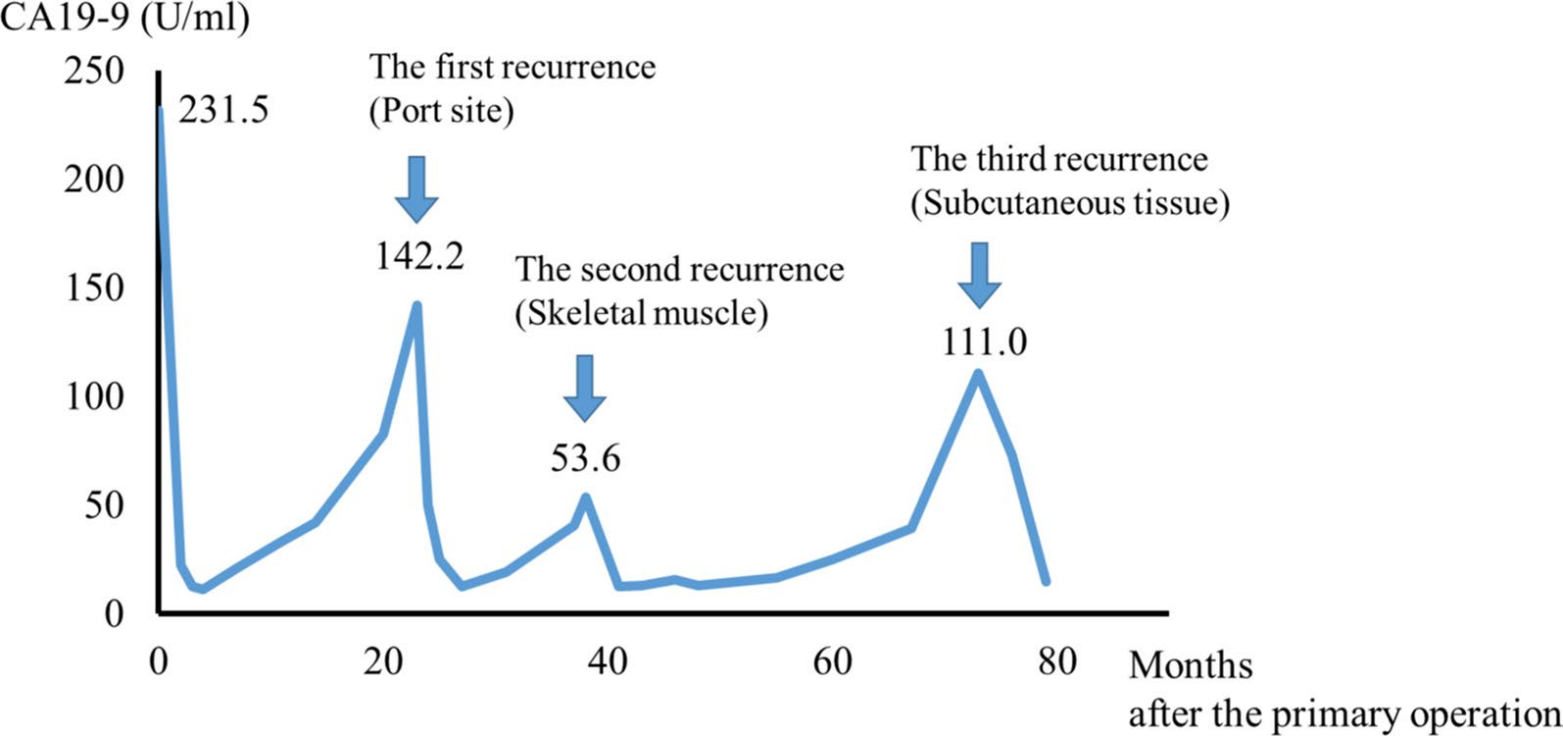
Serum carbohydrate antigen 19-9 (CA19-9) level after the primary operation. Serum CA19-9 level increased according to each relapse of gastric cancer. It responded to each surgical treatment

**Fig. 2:**
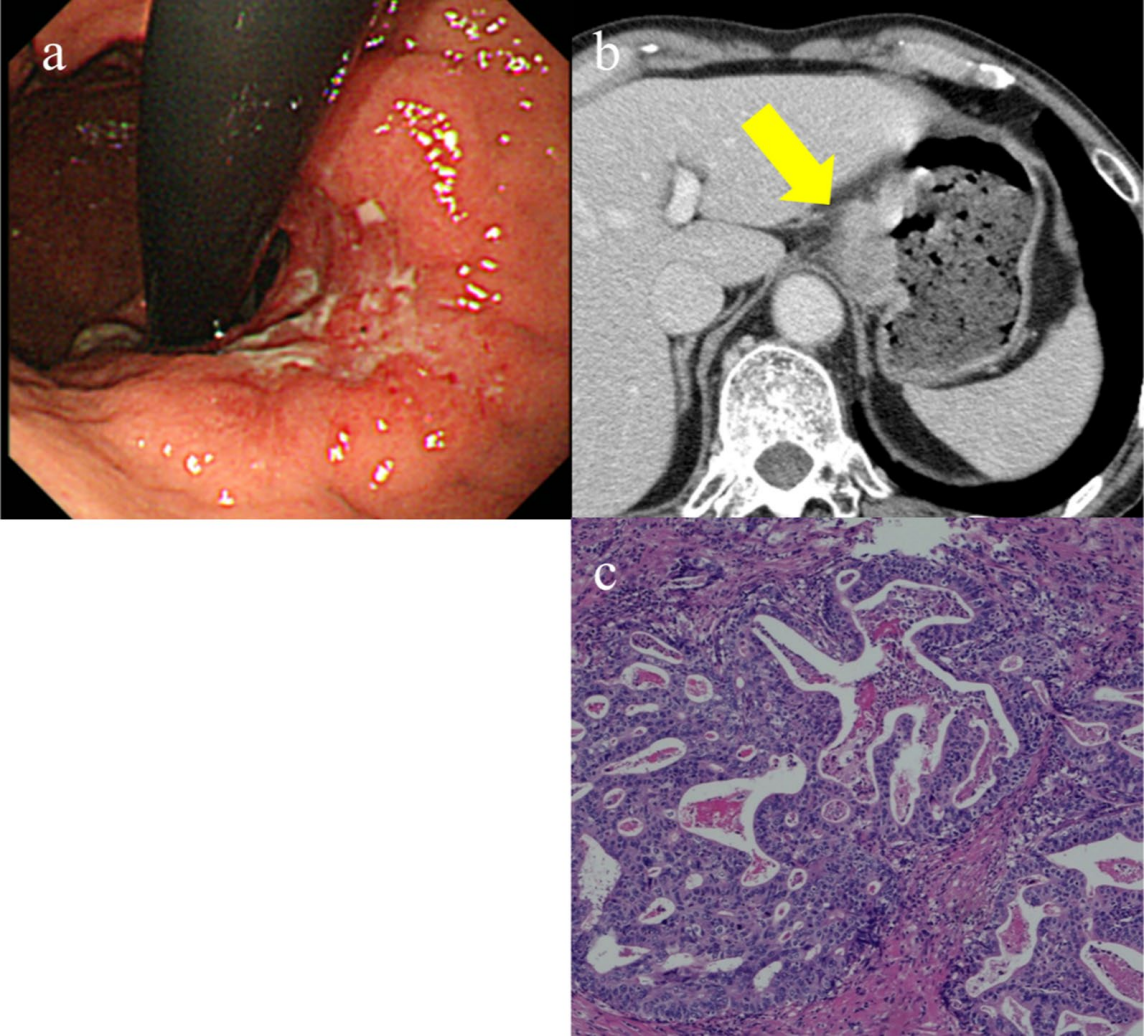
Computed tomography (CT) scan and pathological findings of the primary gastric cancer. **a** Upper endoscopy demonstrated circumferential ulcerated lesion at the cardia. **b** CT scan showing wall thickening at the lesser curvature side of the upper gastric body. **c** Pathological examination reveals a moderately differentiated adenocarcinoma

**Fig. 3:**
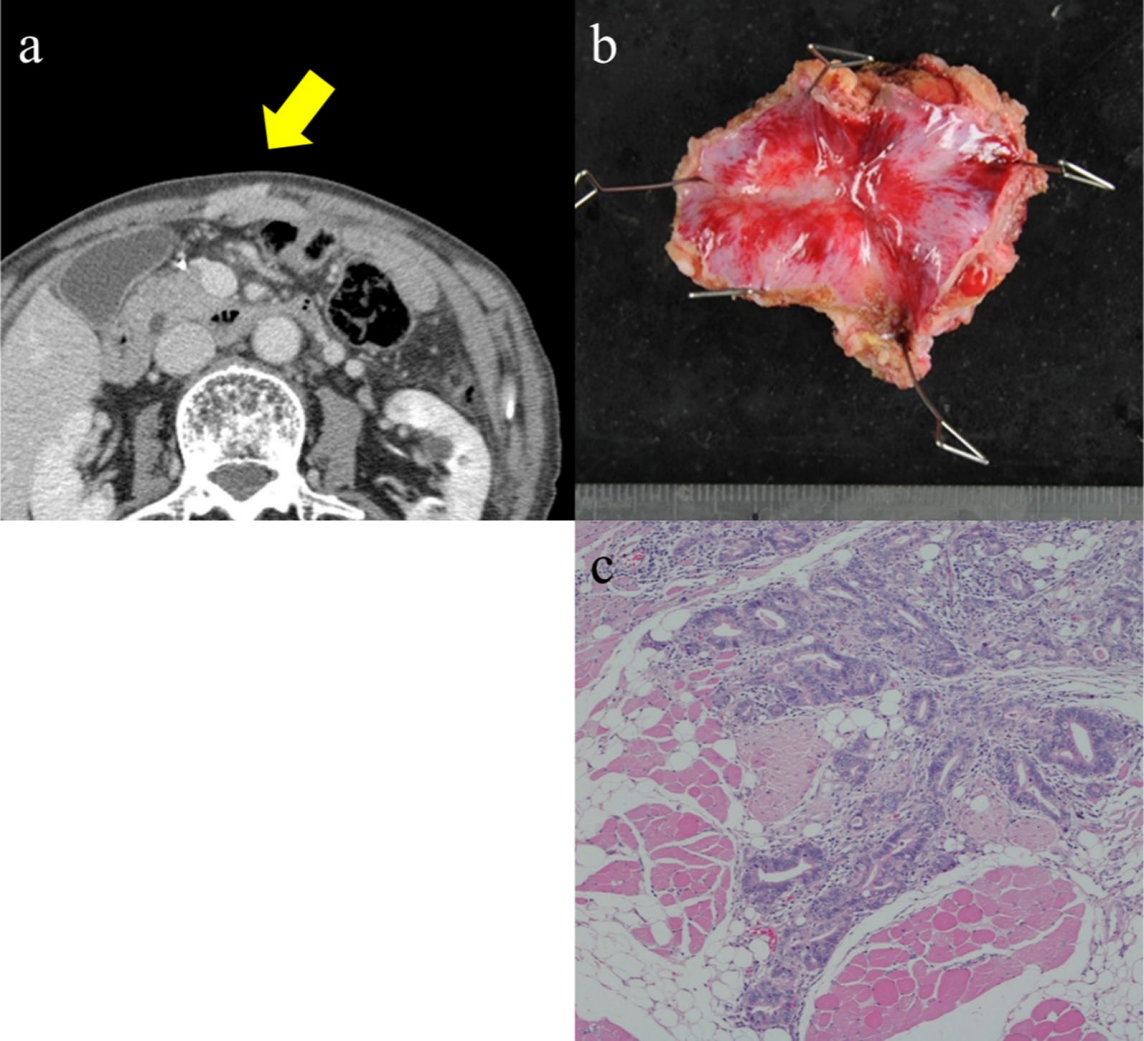
Computed tomography (CT) scan and pathological findings of the first recurrence. **a** CT scan shows an enhanced mass in the median line of abdominal wall. **b** Macroscopic findings show a white nodular lesion in the peritoneum. **c** Pathological examination reveals an adenocarcinoma consistent with metastatic tumor from gastric cancer

**Fig. 4:**
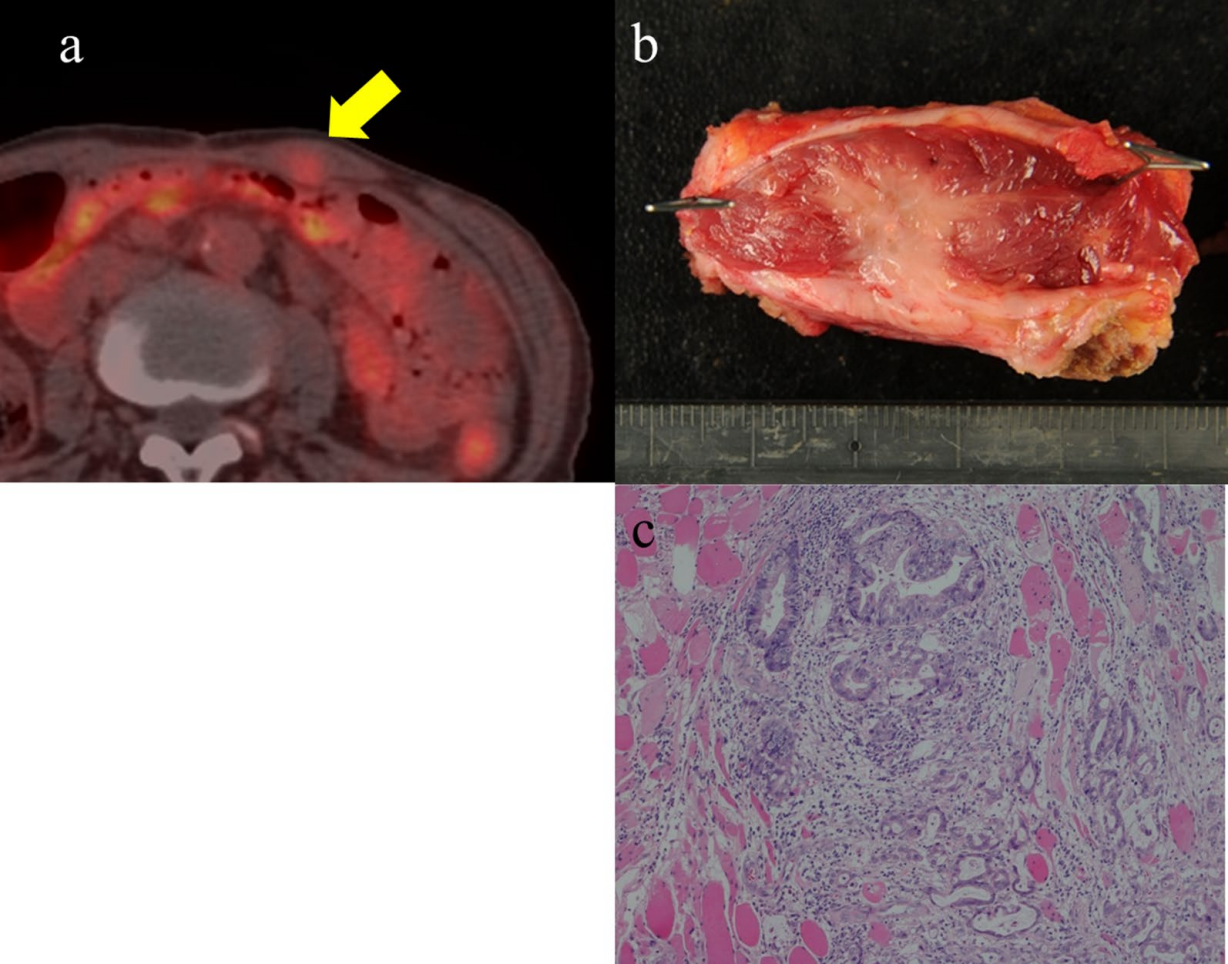
Computed tomography (CT) scan and pathological findings of the second recurrence. **a** Positron emission tomography (PET)/CT scan showing an elevated fluorodeoxyglucose (FDG) intake in the left rectus abdominis muscle. **b** Macroscopic findings showing a white nodular lesion in the skeletal muscle. **c** Pathological examination revealing an adenocarcinoma consistent with metastatic tumor from gastric cancer

**Fig. 5:**
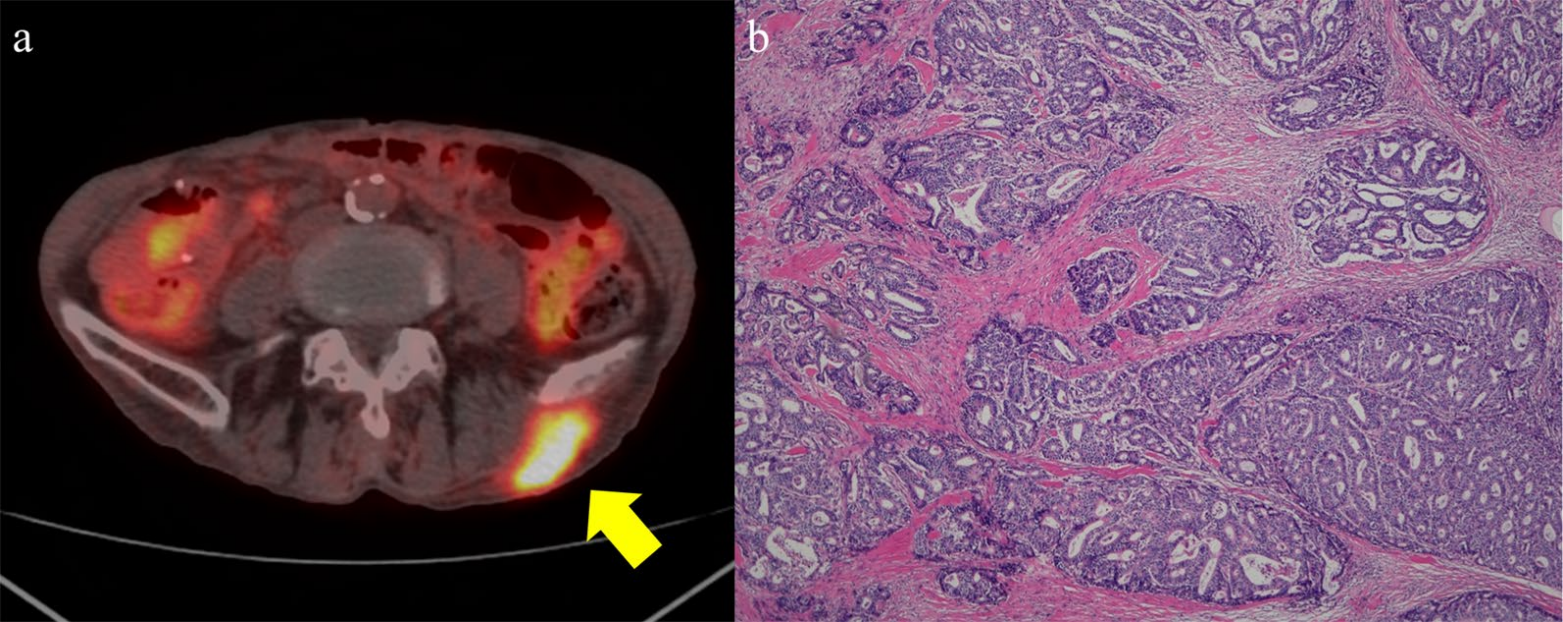
Computed tomography (CT) scan and pathological findings of the third recurrence. **a** Positron emission tomography (PET)/CT scan shows an elevated FDG intake in the lateral area of the left iliocostalis lumborum muscle. **b** Pathological examination reveals an adenocarcinoma consistent with metastatic tumor from gastric cancer

A 75-year-old man was diagnosed with gastric cancer through a medical check-up and was referred to our department. The patient had no subjective symptoms or remarkable past medical history. The tumor marker carbohydrate antigen 19-9 (CA19-9) was elevated, with a value of 231.5 U/ml (Fig. 1). Upper endoscopy demonstrated a 5-cm circumferential ulcerated lesion at the cardia (Fig. 2a). Biopsy showed a poorly differentiated tubular adenocarcinoma. Computed tomography (CT) showed a wall thickening of the lesser curvature side of the upper gastric body (Fig. 2b). The patient underwent laparoscopic total gastrectomy with lymph node dissection, and the pathological diagnosis was consistent with a moderately differentiated tubular adenocarcinoma, pathological stage T4aN1M0, and IIIA according to the UICC classification (Fig. 2c). The postoperative course was unremarkable, and the patient was discharged. He refused adjuvant chemotherapy and was under close observation. The patient was regularly followed up with laboratory tests and imaging studies. Twenty-three months after the primary gastrectomy, a CT scan revealed an irregular mass near the port site wounds (Fig. 3a). The mass continued growing over time, and port site recurrence was suspected. The CA19-9 level increased to 142.2 U/ml (Fig. 1). The patient underwent mass resection, and the pathological diagnosis was consistent with metastatic adenocarcinoma in the subcutaneous tissue at the port site (Fig. 3b, c). In the operative findings, there were no ascites and disseminated nodules in the abdominal cavity. Macroscopic findings of the resected specimen revealed that the center of the tumor was not in the peritoneum, but the abdominal wall. Therefore, it was considered to be a PSM rather than peritoneal dissemination. Thirteen months after the second surgery, CT revealed an enhanced mass in the abdominal wall. Furthermore, PET-CT showed an elevated uptake in the rectus abdominis muscle and a SUV of 3.1 (Fig. 4a). Fine-needle aspiration biopsy of the lesion detected malignant cells with suspected metastatic adenocarcinoma. The CA19-9 level was elevated to 53.6 U/ml again (Fig. 1). The patient underwent mass resection again. The mass had macroscopically infiltrated into the rectus abdominis muscle (Fig. 4b). Similar to the first recurrence, there were no ascites or disseminated nodules in the abdominal cavity. The pathological diagnosis was identical to that of a gastric metastatic adenocarcinoma in the rectus abdominis muscle (Fig. 4c). After thirty-five months from the third surgery, CT revealed a mass in the left gluteal region. PET-CT revealed a 35-mm mass in the lateral subcutaneous area of the left iliocostalis lumborum muscle, which showed an elevated SUV of 9.6 (Fig. 5a). Percutaneous biopsy of the lesion revealed a metastatic adenocarcinoma, and the CA19-9 level was 111 U/ml (Fig. 1). Another mass resection procedure was performed, and the pathological diagnosis was consistent with subcutaneous metastasis from the gastric adenocarcinoma (Fig. 5b). Since tumor cells were present at the resection margin, additional radiation therapy was performed. After each recurrence, the patient did not undergo adjuvant chemotherapy. The patient has survived 78 months after primary gastrectomy.

## Discussion

PSM after laparoscopic gastrectomy has rarely been reported. Previous reports indicate that PSM incidence after laparoscopic gastrectomy for gastric cancer ranges from 0.4% to 11% [4–6]. However, the development of PSM after curative gastrectomy is particularly rare. Curet reviewed PSM etiology and mentioned suspected cause as direct implantation, contamination of surgical instruments, aerosolization of tumor cells, chimney effect, surgical technique, excessive manipulation of tumor, hematogenous spread, and local and systemic effects of carbon dioxide with pneumoperitoneum. Especially, pneumoperitoneum and contamination by inadequate handling of the tumor were two main factors suggested as the cause of PSM [7]. We reviewed previously reported three PSM cases from gastric cancer after laparoscopic gastrectomy (Table 1) [8–10]. The median age was 75 years (range 58–78 years) and all patients were male. The median time since the primary operation was 15 months (range 6–23 months). All patients underwent surgical excision and had a survival duration longer than 6 months. Kim SH et al. reported most PSMs are accompanied by other metastatic lesions, such as lymph-node metastasis or carcinomatosis. On the other hand, PSM can occur even in patients with early stage gastric cancer, like a case reported by Sakurai et al.

**Table 1:** Review of four reported cases of resected PSM from gastric cancer after curative gastrectomy

Case	Author	Year	Age	Sex	Histologic type	Stage	Adjuvant chemotherapy	Time of recurrence after gastrectomy (months)	prognosis after resection of PSM (months)	
1	Lee	2007	73	M	Por	IIIB	5-FU, cisplatin	12	6	N/A
2	Sakurai	2013	58	M	tub2	IA	S-1 (12 months)	18	14	Dead
3	Kim	2015	78	M	por	IIA	None	6	50	Alive
4	Our case	2021	77	M	tub2 > por	IIIA	None	23	55	Alive

*M* male, *por* poorly differentiated adenocarcinoma, *tub2* moderately differentiated adenocarcinoma, *5-FU* 5-fluorouracil, *N/A* not available

The reported incidence of skeletal muscle metastases ranges from 0.03 to 0.16% in clinical practice and 0.8% in an autopsy study [11]. Muscular metastasis from gastric cancer is also rare. Haygood et al. reported that only 21 of 332 cases of skeletal muscular metastasis originated from gastric cancer [12]. It remains unclear why muscular metastasis from gastric cancer is rare. It is believed that frequent changes in blood flow, destruction of tumor cells by muscle movement, tumor proliferation inhibition by lactic acid protease, and muscle pH may be protective factors [13].

We reviewed previously reported cases of muscular metastases from gastric cancer. We searched the PubMed database using the keywords of “gastric cancer,” “muscle metastasis,” and “muscular metastasis”. We found six muscular metastases cases after curative gastrectomy (Table 2) [14–19]. The median age was 61 years (range 47–82 years) and all patients were male. The median time since the primary operation was 15 months (range 7–74 months). In general, skeletal muscle metastasis from gastric cancer is considered to be a wide-spread metastatic disease and carries a poor prognosis. The prognosis of reported cases was also poor. Only our patient remained alive for more than 3 years after recurrence. Two solitary metastatic cases, including that of our patient, underwent surgical excision. Surgical excision was also performed to relieve the pain from the metastatic tumor. Kamitani reported that a skeletal muscle metastasis without metastasis to any other organ tends to occur in patients with relatively early stage disease and undifferentiated carcinoma [19]. In our review of previous articles, four out of seven cases were undifferentiated carcinoma. Regarding the tumor progression, except for our patient, all of reported cases whose tumor stages were available were stage I or II. Therefore, we have the same opinion as Kamitani.

**Table 2:** Review of seven reported cases of metachronous skeletal muscle metastasis after curative gastrectomy

Case	Author	Year	Age	Sex	Histologic type	Stage	Adjuvant chemotherapy	Time of recurrence after gastrectomy (months)	No. of metastasis	Treatment	Prognosis after recurrence (months)	
1	Sudo	1993	61	M	Adenocarcinoma	N/A	N/A	60	Solitary	CRT	6	Dead
2	Amano	1996	57	M	Adenocarcinoma	N/A	N/A	7	Multiple	N/A	N/A	Dead
3	Ghanekar	1997	75	M	por	IIA	N/A	15	Solitary	Surgery	1	Alive
4	Beşe	2006	60	M	sig	IIA	5-FU, leucovorin	13	Multiple	CRT	11	Alive
5	Koga	2015	71	M	por	IB	None	36	Multiple	Chemotherapy	1	Dead
6	Kamitani	2018	47	M	por	IIA	S-1	12	Multiple	Chemotherapy	7	Dead
7	Our case	2020	79	M	tub2 > por	IIIA	None	36	Solitary	Surgery	42	Alive

*M* male, *por* poorly differentiated adenocarcinoma, *sig* signet ring cell carcinoma, *tub2* moderately differentiated adenocarcinoma, *N/A* not available, *CRT* chemoradiotherapy

A subcutaneous metastasis is a relatively uncommon manifestation of visceral malignant tumors that occurs more often in the late stages of the disease. Subcutaneous and cutaneous metastases commonly originate from breast cancer. Cancers of the lung, colon or rectum, oral cavity, kidney, and ovary have similar cutaneous metastasis rates, ranging from 3.4% to 4% [20]. The reported incidence of subcutaneous metastasis from gastric cancer is only 0.8–1.0% [1–3]. Generally, a subcutaneous metastasis from gastric cancer is considered to be an end-stage presentation with a poor prognosis. Most cases of subcutaneous metastasis are indicative of widespread dissemination of the disease. Bordin and Weitzner reported that the duration of survival after the diagnosis of metastatic carcinoma in the skin averaged 11.4 weeks, with a range of 2–34 weeks [21]. This is because the treatment usually consists of systemic or palliative therapy rather than surgery.

We searched the PubMed database for earlier case reports treated by surgical excision of subcutaneous metastasis due to gastric cancer using the key words “gastric cancer,” “cutaneous metastasis,” “subcutaneous metastasis,” and “skin metastasis.” We found six subcutaneous metastasis cases without any other synchronous metastases after curative gastrectomy (Table 3) [22–27]. The median age was 68 years (range 60–89 years). Only one case involved a female patient. The median time to recurrence after the primary operation was 71 months (range 4–120 months). Only two out of the seven cases survived longer than 3 years after resection of the subcutaneous metastasis. This may indicate that surgical resection prolongs survival for isolated subcutaneous metastasis.

**Table 3:** Review of eight seven cases of resected subcutaneous metastasis from gastric cancer after curative gastrectomy

Case	Author	Year	Age	Sex	Histologic type	Stage	Adjuvant chemotherapy	Time of recurrence after gastrectomy (months)	Prognosis after resection of subcutaneous metastasis (months)	
1	Fruh	2005	60	N/A	Adenocarcinoma	II	N/A	24	90	Alive
2	Karayiannakis	2010	76	M	por, sig	III	Irinotecan, L-OHP	4	1	Dead
3	Wang	2014	68	M	adenocarcinoma	I	None	120	8	Dead
4	Cesaretti	2014	60	M	por	III	Epirubicin, CDDP, 5-FU CDDP, capecitabine, trastuzumab	72	40	Alive
5	Morita	2017	65	F	tub1	IIA	N/A	60	18	Alive
6	Koyama	2019	89	M	N/A	IV	S-1	72	N/A	
7	Our case	2020	82	M	tub2 > por	IIIA	None	71	7	Alive

*M* male, *F* female, *N/A* not available, *por* poorly differentiated adenocarcinoma, *sig* signet ring cell carcinoma, *tub2* moderately differentiated adenocarcinoma, *tub1* well-differentiated adenocarcinoma, *L-OHP* oxaliplatin, *5-FU* 5-fluorouracil, *CDDP* cisplatin

Our patient had long-term survival by undergoing surgical resection. It is difficult to clarify indications for surgical treatment for subcutaneous and skeletal muscle recurrence that have poor prognosis. However, the following two points are considered indicators of local control. One is that the recurrent tumor is single. The other is that there is a certain period of time before recurrence. In our review, patients who survived more than 6 months after performing resection of recurrent tumors of muscular and subcutaneous metastases have at least 12 months from the initial surgery. The fact that a certain period of time has passed until recurrence is considered to be an indication of local control, and tumor resection may be considered as a treatment option.

In this report, we described the case of a patient who repeatedly developed recurrence of port site, subcutaneous and muscular metastases from gastric cancer. This patient had an extremely rare presentation for two reasons. First, the patient had a relapse of gastric cancer with the port site, muscular and subcutaneous metastases repeatedly, and all of them are rare manifestations. Second, although both subcutaneous and muscular metastases were associated with poor prognosis, this patient survived for 77 months after the primary gastrectomy.

## Conclusion

Here we report an extremely rare case of repeated port site, muscular and subcutaneous metastasis due to gastric cancer with long-term survival after resection of the metastatic tumor. Generally, the prognosis of both subcutaneous and muscular metastasis is poor. However, if the metastatic tumor is solitary, surgical excision could be a feasible treatment and might prolong survival.

## Data Availability

Not applicable.

## Abbreviations

UICC: Union for International Cancer Control
CT: Computed tomography
PET CT: Positron emission tomography
SUV: Standardized uptake value
PSM: Port site metastasis
CA 19-9: Carbohydrate antigen 19-9
